# Case of Rupture on the Uterus

**Published:** 1855-01

**Authors:** John Neill

**Affiliations:** Surgeon to the Pennsylvania Hospital


Case of Rupture on the Uterus. By John Neill, M. D., Surgeon to the Pennsylvania Hospital.—Monday, 24th of July, called to Mrs. John McDevitt, South above 20th Street, in labor with her sixth child; reached her at 9 P. M.; had been in active labor for about an hour; of a florid complexion, somewhat fleshy, large muscular development, with all the appearance of possessing a constitution of more than ordinary strength and vigor.

Upon examination, found the os uteri about half open, membranes presenting unbroken, the head to be felt high up above the superior strait; the pains were good, but by no means violent, with distinct remissions of five or six minutes; left her, and returning in about an hour, found the os uteri fully open, pains somewhat stronger, but still the presenting part did not descend. I now ruptured the membranes in the expectation that as there was neither contraction nor rigidity, the head would come down into the pelvis without further delay. In this I was disappointed, and it was evident that the head had some difficulty in entering the superior strait. Still it advanced a little, and I thought I could detect the anterior fontanel looking towards the left acetabulum, giving the fourth position of Baudelocque. I was however by no means certain on this point, but resolved to wait. I made a visit in the next street and returned to the patient in less than half an hour; the pains were now much stronger, and I thought that the head had advanced slightly. At this time Mrs., M. was obliged to get up, for the purpose of relieving her bowels, and I went down stairs, still without the slightest anxiety as to the result of the labor, for the spirits were good, the countenance cheerful, the woman well formed and vigorous, and a state of active labor had not existed for more than two hours and a half.

While at stool, the patient had two pains; during the latter she suddenly complained of intense agony, with a burning sensation in the right side; the woman hurried her to bed, and called me into the room; I found her on her back in great torture, which she assured me was no longer the pain of labor, but that something had gone wrong inside of her.

I examined her pulse and found it but little altered; this, added to the circumstance of there being neither vomiting nor cold clammy skin, nor anything like an approach to syncope made me hope that matters were not so bad as I had at first apprehended; but after administering some forty drops of laudanum, using hot fomentations, and waiting for some time, finding that the uterine contractions were completely suspended, that the presenting part had receded, and that there was a sanguineous discharge, though not profuse, from the vagina, I felt convinced that the uterus was ruptured.

Before, however, proposing any operation, I called upon Dr.

Hollingsworth for his advice and assistance. He immediately came in the kindest manner, and after careful investigation, the diagnosis was distinctly made out. The placenta had not passed into the cavity of the abdomen, for it could be distinctly felt with the cord passing from it. The head of the child could be detected through the abdominal parietes occupying the lower part of the abdomen on the right side, near the inguinal region, but no portion of it remained in the uterus.

Under these desperate circumstances, we deliberated with sad forebodings on the treatment to be adopted, in order, if possible, not to lessen the little chance of life remaining. I am aware that all the best authorities recommend introducing the hand through the torn womb, into the bowels of the victim, seizing the feet, and first dragging the infant back into the womb, and from thence, per vias naturales, into the world. They say that it gives the child a better chance; it may be so, I am not prepared to dispute that point, for I thank God I have had no experience in such a procedure; but this I do know, that the description of the operation has always filled me with unutterable horror.

Another thing, which I am bound to confess, though it may appear very unprofessional and unnatural to some, is, that I never thought of the child or its life, or anything about it, except to wish, that as it had pleased God to place it there, it would please him, in his infinite mercy, to assist me in getting it away, without tearing the poor devoted woman to pieces, and entailing upon myself the terrible conviction, that I had made almost certain death a certainty.

After due consideration, however, we determined to explain the nature of the necessary operation, by turning to the patient, and propose it as a dernier resort. This we did, but she absolutely refused to submit to it; and, from the hydrocephalic condition of the head, afterwards ascertained, we had reason to be thankful that she did so. At this time there was no vomiting, the expression of her countenance was good, the pulse firm, and the skin natural; the pain in the abdomen, at first very severe, had now much abated; and aftering administering a powerful dose of morphia we left her, determined to see her in the morning, and then be guided by circumstances.

Next morning, the 25th, when Dr. Hollingsworth and myself visited her, we found her much better than we anticipated; pulse firm and strong, countenance bright and mind unclouded; longer to leave her undelivered was out of the question; professional duty and common humanity alike demanded that an effort, however desperate, should be made to save her. We, therefore, determined to propose gastrotomy, as that operation, in our opinion, afforded her the best chance. To this, encouraged by feeling better than she anticipated, she at last consented ; and, after consulting Dr. Neill, who undertook the performance of the operation, it was determined on.

*Operation by Dr. Neill.*—The patient was placed upon a stout table covered with blankets, her shoulders and head supported by pillows ; and, as a preliminary step, about four ounces of ether were administered by inhalation. The incision was made in the linea alba, commencing about two inches below the umbilicus, and extending towards the pubis full six inches. The moment the opening was made, large quantities of mingled blood and clots escaped, the omentum seeming saturated with blood, and both the visceral and parietal peritoneum being deeply stained. A dead child's back presented, its head lying low down towards the right groin, its feet to the left. It was immediately removed, and found to be hydrocephalic ; the bi-parietal diameter of the head measuring, I should suppose, six inches, the occipito frontal probably seven. Its entire weight I should judge to be not less than ten pounds.

The rent in the uterus appeared to be enormous, and perfectly uncontracted, for the operator passed both hands through it, right down into the organ, and, as it were, scooped up the placenta, with all the coagula within his reach. Upon the removal of his hands, the womb instantly contracted to about the size of a man's fist. The blood, fluid as well as coagulated, was then removed from the cavity of the abdomen as far as practicable, disturbing the viscera as little as possible. The incision was then closed by five sutures, and afterwards by long adhesive straps, leaving an opening at the lower part of the wound to favor the escape of fluids ; a compress and binder completed the arrangement.

The patient's strength was less exhausted than could have been anticipated; spirits good ; pulse 120—firm and equal. The time occupied by the whole operation did not, I should think, exceed five minutes. In half an hour she was placed in bed, and an enema of laudanum administered ; grain doses of opium were directed to be given by the mouth every three hours, in order to keep her, if possible, in a perfect state of repose, and prevent any action of the abdominal viscera ; at the same time the system was supported by nourishing fluids, beef-tea, &c.

Shortly after the operation, the patient began to vomit a greenish watery fluid, which continued several hours, but was checked by the exhibition of small quantities of brandy with ice ; the opium treatment seemed to agree with her, for she slept, and complained of but little pain.

On the morning of the 26th I visited her in company with Dr. Hollingsworth. Found her tolerably easy ; mind cheerful ; tongue clean and moist ; pulse 120 ; the abdomen was tympanitic, and very much distended ; the breathing much embarrassed by the accumulation of gas. Ordered her to continue the opium pills, and to have an injection containing turpentine.

On making my evening visit I learned that the bowels had been slightly opened, and that she had passed large quantities of flatus, by which the tympanitic distension of the abdomen was much lessened; breathing easy and natural; pulse rapid and weak. Directed brandy to be continued with the opium.

27th and 28th.—Continued in much the same condition—occasionally vomiting.

On the 29th Dr. Neill visited her with Dr. Hollingsworth and myself. Removed the stitches from the wound, which was healthy and closing extremely well; she was now ordered milk punch, ad libitum.

At this time there was a very copious, dark, offensive discharge from the vagina, which was kept continually syringed with warm water and soap. The bowels had been moved once copiously; pulse 120; tongue moist, but slightly furred. The patient looked so hopeful and strong, that we began to feel encouraged.

On the morning of the 30th I understood that she had passed a restless night. Looked very much wore; the lips were pale; countenance dejected; pulse 130; vomiting of green matter without effort, in fact a regurgitation of the fluids contained in the stomach.

I began to lose hope. Still her mind never wavered—day nor night—and when spoken to she replied quickly and clearly, but without anything like an unnatural elevation, a condition which I have sometimes observed in bad cases of uterine phlebitis. The discharge from the vagina was less copious and less offensive.

When I saw her in the evening, she was laboring under the worst possible symptoms; so much so, indeed, that I thought it possible she might die before the morning. Her pulse was from 135 to 140—very weak; her feet and legs were cold, also the lower part of the belly; her wrist and arms to the shoulders in the same condition, and bedewed with a clammy sweat.

I confess I regarded her as moribund, and the priest in attendance told her that she was dying, and must make her peace with God; the poor woman replied that she would make her peace with God most willingly, but that the rev. father was wrong, that she was not dying yet, she did not feel like dying. And as it proved, she was right, for the next morning, the 31st, I was agreeably surprised to find that her skin had regained its natural temperature, that her strength had improved, and that she was altogether better than on the previous day. There was no pain on pressure of the abdomen, but still there was considerable tympanites, and the pulse continued at 135.

On the first of August the vomiting continued, but only occasionally.

On the 2d, vomiting had ceased entirely. I watched with great anxiety for a diminution in the frequency of the pulse, as indicating some favorable change, but as yet in vain.

Notwithstanding the steady pursuance of the opiate treatment, the patient's bowels, on the 1st, were largely opened, three or four times. She complained of great pain, before each evacuation; opiate enemata checked this, and on the second she had but one stool, perfectly natural in color, and of the consistence ordinarily produced by a dose of castor oil.

*August* 3. Pulse somewat slower—about 120; dressed the wound in the abdomen; did not think it looked quite so well; some discharge from one of the suture openings: was suffering from great uneasiness of the bowels, they having been opened several times; before each movement, considerable pain was complained of, somewhat resembling the tormina of dysentery; color perfectly natural. In the evening, there again appeared great coldness of the extremities. Ordered an enema of starch and laudanum, with hot bricks to the legs and feet, brandy and milk to be given freely.

On the 4th, found the patient warm; pulse 130; tongue foul; complaining of great pain in the bowels; which had been moved several times during the night; the breathing high and labored. Both Dr. Neill and myself thought her prospect of recovery worse than usual. Dr. Hollingsworth had left town, and I was obliged to be absent from the city for some hours; Dr. Neill, therefore, undertook to see her for me. In the evening, I found her symptoms the same as in the morning. Ordered her a large teaspoonful of laudanum, as an injection, and desired the attendant to give her all the nourishment she could take, with a continuance of the brandy and milk.

Saw her, with Dr. Neill, on the morning of the 5th. Breathing decidedly improved; countenance and spirits better, though the pulse was weak and continued at 130; tongue cleaning; dressed the abdominal wound, which looked much healthier, though there was considerable discharge from another of the suture wounds; had passed a comfortable night, slept well, and had had no pain nor trouble with her bowels; but there had been a considerable discharge from the womb, described by the nurse as being of a clear red color, and devoid of smell.

At half past nine in the evening, saw her again. Condition unchanged; pulse the same; uterine discharge still copious; bowels had been opened once.

On the 6th, found her much improved; pulse 120; tongue clean; expression of face natural; heat of skin almost natural, with very little thirst; the abdominal wound nearly healed. In the evening, found her easy, but showing more weakness. This I attributed to the uterine discharge, which continued copious.

Morning of the 7th, stronger and better; pulse 110; discharge from the womb much lessened; had slept soundly all night. At ten o'clock, evening of the same day, great change had taken place; pulse 100, firm and steady; uterine discharge nearly suppressed; had taken her food regularly, and with appetite; bowels had been opened once naturally during the day.

From this time, she improved so rapidly that, on the 15th, she came down stairs; on the 24th, just a month from the time of the rupture, was at the washtub; and, on the 3d of September, I met her in the street, when she told me she felt as well as she did before the accident.

