# Correction

**Published:** 1855-03

**Authors:** 


### CORRECTION.

In the Record Department of the January number of this Journal, Dr. Mason's paper, entitled "Case of Rupture of the Uterus," &c., is incorrectly credited to the operator, Dr. John Neill. Through oversight in our compositor the usual credit was not given to our respected cotemporary, the *Medical Examiner*, from which the article was copied.

